# Redox Status and Hematological Variables in Melatonin-Treated Ewes during Early Pregnancy under Heat Stress

**DOI:** 10.3390/vetsci9090499

**Published:** 2022-09-13

**Authors:** Efterpi V. Bouroutzika, Ekaterini K. Theodosiadou, Mariana S. Barbagianni, Serafeim Papadopoulos, Dimitrios Kalogiannis, Stella Chadio, Zoi Skaperda, Demetrios Kouretas, Eleni G. Katsogiannou, Irene Valasi

**Affiliations:** 1 Faculty of Veterinary Science, University of Thessaly, 43131 Karditsa, Greece; 2 Department of Ichthyology and Aquatic Environment, University of Thessaly, 38446 Volos, Greece; 3 Department of Animal Science, Agricultural University of Athens, 11855 Athens, Greece; 4 Department of Biochemistry and Biotechnology, University of Thessaly, Viopolis, Mezourlo, 41500 Larissa, Greece

**Keywords:** ewes, heat stress, melatonin, fertility rate, total antioxidant capacity, lipid peroxidation, glutathione, cortisol, hematological variables

## Abstract

**Simple Summary:**

Heat stress induces oxidative stress that negatively affects the fertility rate in farm animals, as it may disrupt normal follicular development and preimplantation embryogenesis. Although indigenous sheep breeds may exhibit adaptation to a certain level in high summer temperatures, their reproductive competence is still compromised under extreme severe heat stress conditions. Thus, the administration of melatonin is suggested, as an antioxidant regime, for improving the fertility rate and redox balance in heat-stressed ewes. The influence of environmental thermal stress in conjunction with melatonin treatment on hematological variables and cortisol secretion was also investigated. In this respect, melatonin seems to enhance the progressive adaptation of indigenous-breed ewes to environmental heat stress.

**Abstract:**

The preovulatory follicles and preimplantation stage embryos are found to be rather sensitive to heat stress due to their low potential for scavenging reactive oxygen species (ROS). The aim of the present study was to assess the impact of melatonin administration on redox status and hematological variables during the preovulatory period and early stages of embryogenesis in heat-stressed ewes in vivo. Forty Karagouniko-breed ewes were divided in two groups, the melatonin (M, *n* = 20) group and control (C, *n* = 20) one. All animals were subjected to heat stress throughout the study, which lasted forty days (D0 to D40). In M group, melatonin implants were administered on D0. Then, oestrous synchronization was applied (D19-D33). On D34, six rams were introduced into the ewe flock for mating. Ultrasonographic examination was conducted on D73 for pregnancy diagnosis. The temperature humidity index (THI), the rectal temperature (RT), and the number of breaths per minute (BR) were evaluated twice daily. Redox biomarkers, namely total antioxidant capacity (TAC), reduced glutathione (GSH), and thiobarbituric acid reactive substances (TBARS), were assayed in blood samples collected on D0, D33, and D40. In addition, packed cell volume (PCV), white blood cells (WBCs), leukocyte differential count, and cortisol assessment were conducted in blood samples on D33 and D40. The results indicated improved fertility rate and mean number of lambs born per ewe due to improved redox status (*p* < 0.05) in ewes that received melatonin implants 34 days approximately before the onset of oestrus. The PCV decreased in both groups between the two time-points (*p* < 0.05). However, the NEU/LYMPH ratio decreased (*p* < 0.05) only in group M. The low cortisol levels and the decreased NEU/LYMPH ratio in both groups support the hypothesis that ewes of the indigenous Karagouniko breed may exhibit adaptation to environmental thermal stress. The administration of melatonin as an antioxidant regime may improve the reproductive competence of heat stressed ewes and may also enhance their ability to adapt at high ambient temperatures.

## 1. Introduction

Heat waves impose heat stress on mammals [1], negatively affecting their reproduction and production. Small ruminants belong among the domestic species intensively affected by heat stress, since in regions such as the Mediterranean area, are usually reared in extensive and semi-intensive production systems.

To maintain the welfare of sheep, is critical to determine the thermoneutral zone, which ranges from 20 to 30 °C. Every increase of temperature above 34 °C can compromise the welfare and even the animals’ survival [2,3]. It is well known that stress activates the hypothalamo–pituitary–adrenal axis and the sympathoadrenal system, with end result the secretion of glucocorticoids and catecholamines for relieving the stress effects [4]. However, adrenocorticotropic hormone (ACTH) stimulated release of glucocorticoids causes an inhibitory effect on the reproductive axis. Apart from that, under stress conditions a number of hematological variables as well as physiological and behavioral traits are differently expressed, such as changes in breathing and heart rate, ruminal motility, body temperature, and sweating [5].

Reproduction is a complex process with hormones, metabolites and environmental signals interacting alongside. Almost all the reproductive processes, from the stage of follicular growth and oocyte maturation to parturition, as well as lactation and uterus involution, could be affected by heat stress. However, in farm animals the principal adverse effect of heat stress on reproduction is the low fertility rate, mainly due to high susceptibility of preovulatory follicles and of preimplantation stage embryos to high maternal temperature [6,7]. The early developmental stages from oocytes to cleavage-stage are thermosensitive, while advanced developmental stages of early embryos, such as morulae and blastocysts are thermotolerant. During these developmental stages, thermosensitivity is related to increased oxidative stress levels [7]. Accumulating evidence indicate that ROS and antioxidant systems participate in reproductive processes, such as follicular growth, ovulation, fertilization, luteal steroidogenesis, implantation and developmental competence of embryo(s) [8]. Although ROS production promotes embryo(s) implantation, an uncontrolled ROS accumulation could lead to early embryonic loss [8]. Thus, side-effects caused by elevated temperatures can be remedied with administration of antioxidants, as has been suggested by in vivo [9,10] and in vitro studies [11].

Melatonin is an indoleamine with anti-inflammatory and antioxidant properties acting as a scavenger for free radical species [12,13,14,15]. It exerts pleiotropic bioactivity and effectively synchronizes various physiological and pathological conditions, such as maternal-fetus unit and circadian rhythms stimulated by the photoperiod [16], immune-response, body weight gain and reproductive processes [17]. Regarding the reproductive processes in sheep, melatonin has been shown to be involved in follicular development and luteal function [18,19,20] or in embryonic development in vitro [20,21]. The most well characterized effects of melatonin administration under field conditions in sheep, concern its role in accelerating the onset of the reproductive season [22] and improving litter size and pregnancy rates in anoestrous ewes [23,24]. However, a recent study of our team provided evidence that this hormone can be safely administered throughout gestation, leading to enhanced redox profile of heat-stressed ewes, higher mean number and bodyweight of lambs born per ewe and better milk yield [25]. Similarly, melatonin treatment resulted in better onset of cyclicity by improving the antioxidant profile and lessening the oxidative stress in summer heat-stressed anoestrous buffaloes [26].

Concerning the low potential of oocytes and preimplantation embryos for scavenging ROS under heat stress conditions, this study was designed to assess the effect of melatonin administration on redox status, hematological variables and cortisol levels during the preovulatory and early embryonic period in heat-stressed ewes. For this purpose, melatonin implants were administered in ewes 34 days before the onset of induced oestruses, in order to ensure that melatonin concentration in blood circulation was at maximum level.

## 2. Materials and Methods

### 2.1. Animals and Treatments

In total, 40 ewes of Karagouniko breed, ranging from 2 to 4 years old and 3.00 to 3.50 body score condition, were included in the study. The study was performed during the breeding season (lasting from June to February,) at summer in central Greece (Karditsa, Thessaly; 21°55′17′′ Ν/39°21′50′′ Ε). Ewes were divided in two groups, the melatonin (M, *n* = 20) group and control (C, *n* = 20) one. The study period started on the day (D0) of the administration of melatonin implants to ewes of group M, subcutaneously at the base of the ear (dose rate: 1 implant per ewe; Regulin, Ceva, Lisbourne, France [22]. Then oestrous synchronization was applied with intravaginal insertion of progestogen sponges in ewes of both groups (flugestone acetate 20 mg; Chronogest, Intervet, Boxmeer, The Netherlands) from D19 to D33, followed by an intramuscular eCG injection on D33, when the sponges were removed (dose rate: 300 UI; Gonaser, Laboratorios Hipra, Girona, Spain). On D34, six rams of known fertility, fitted with crayon-equipped marking harnesses were introduced into the ewe flock until D40, for marking ewes that accepted mating. On D73, ultrasonographic examination was performed in all ewes for pregnancy diagnosis. Finally, the number of lambs born per ewe was recorded at parturition. Ewes were fed with 300 g of ratio twice a day, 1 kg clover and 2 kg alfalfa hay daily, and had access to water *ad libitum*. After the 100th day of pregnancy, ewes that bore one embryo were fed with 350 g of ratio twice a day and ewes that bore more embryos were fed with 400 g twice a day; all animals were fed 1.5 kg of clover and 2 kg of alfalfa hay daily.

Ewes were fully housed from 9.00 am until 6.00 am next day. During those three hours the animals were grazing the surrounding pasture. From D0 until D40, the temperature and humidity were recorded outside and within the animals’ facilities, for six hours every day (12.00–18.00 h) by using a portable temperature data logger, placed on a table (HD 32.2, Delta OHM, Caslle di Selvazzano, Italy).

Body temperature (°C) was measured by a rectal digital thermometer, and breathing rate (breaths per minute, bpm) was estimated by counting flank movements, in ten ewes in each group (total 20 ewes), twice daily (12.00–18.00 h) from D34 until D40. Collection of blood samples was performed in the same 20 ewes by jugular venipuncture in tubes containing EDTA, before the administration of melatonin (D0), then at removal of sponges (D33) and 7 days later (D40). The time point of D40 was chosen by estimating approximately the time of morulae formation (4 days after ovulation), based on the timing of pre-attachment embryogenesis [27].

**Figure 1:**
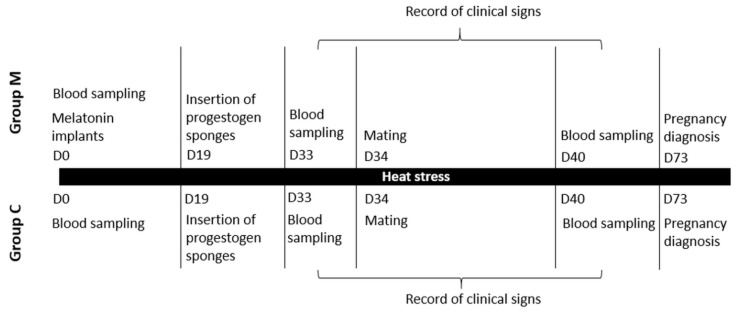
Experimental overview of the study.

The experimental overview is depicted in Figure 1.

### 2.2. Laboratory Assays

#### 2.2.1. Hematological Variables

In blood samples collected on D33 and D40, the microhematocrit method was used for estimating the packed cell volume (PCV) value [28]; the height of the red column was measured after centrifugation of tubes. Based on a previous study [29], the assessment of leukocytes (white blood cells (WBCs), neutrophils (NEU), lymphocytes (LYMPH), monocytes (MONO), eosinophyls (EOS)) and platelets (PLTs) counts was performed by means of blood smears stained with Giemsa staining for each blood sample separately. A leukocyte differential count (200-cells) was also calculated microscopically and then, the neutrophils per lymphocytes (NEU/LYMPH) ratio were estimated.

#### 2.2.2. Cortisol Measurement

Cortisol concentration was determined in plasma samples, collected on D33 and D40, by means of a ^125^I radio-immunoassay kit (Cortisol RIA CT, KIPI28000, DIAsource ImmunoAssays S.A., Belgium) for γ counter. The inter- and intra-assay precision was 11.5% and 5.2%, respectively.

#### 2.2.3. Redox Status Biomarkers

For assaying redox status biomarkers, blood samples collected at three time points (D0, D33, and D40) were properly handled after collection and stored at −20 °C. Within three months the data were retrieved in triplicate.

Total antioxidant capacity (TAC) assay was based on Janaszewska and Bartosz [30] as previously described [31]. Twenty μL of plasma sample were incubated with 10 mM sodium phosphate buffer pH = 7.4 (480 μL) and 0.1 mM 2,2-diphenyl-1-picrylhydrazyl radical (DPPH•) solution (500 μL) in the dark for 1 h at 20 °C, centrifuged (20,000 g, 3 min, 4 °C) and the optical density was estimated at 520 nm using spectrophotometer (U-1900; Hitachi, Ltd., Tokyo, Japan). TAC was calculated on the basis of the mmol DPPH• reduced by the antioxidants present in the samples.

Reduced glutathione (GSH) assay was based on Veskoukis [32]. Twenty μL of erythrocyte lysate treated with TCA were incubated in 67 mM phosphate buffer (pH = 7.95) (660 μL) and 1 mM 5. 5-dithiobis (2 nitrobenzoic acid) (DTNB) (30 μL), for 45 min in the dark at 20 °C. The optical density was estimated at 412 nm using spectrophotometer (U-1900; Hitachi, Ltd., Tokyo, Japan). GSH concentration was calculated on the basis of the millimolar extinction coefficient of DTNB (13.6 L/mmoL/cm). Hemoglobin concentration of erythrocyte lysate was measured using a commercially available kit.

Thiobarbituric acid reactive substances (TBARS) assay was based on Veskoukis [32]. One hundred μL of plasma were added in 35% trichloroacetic acid (TCA) (500 μL) and 200 mM Tris-HCl pH = 7.4 (500 μL) and incubated for 10 min at 20 °C. Afterwards, 1000 μL of 2 M Na_2_SO_4_ and 55 mM of thiobarbituric acid (TBA) were added in the primary solution. Following 45 min incubation at 95 °C, 1000 μL of 70% TCA were added and then centrifuged (15,000× *g*, 3 min, 20 °C). The optical density was estimated at 520 nm using spectrophotometer (U-1900; Hitachi, Ltd., Tokyo, Japan). The concentration of TBARS was calculated on the basis of the millimolar extinction coefficient of malonyldialdehyde (156 L/mmol/cm).

### 2.3. Data Management and Analysis

#### 2.3.1. Temperature—Humidity Index

The Temperature—Humidity Index (THI) was calculated according to Marai et al. [6] and the below equation was used: THI = db °C − [(0.31 − 0.31 × RH) × (db °C − 14.4)], in which: db °C was the ‘dry bulb’ temperature provided by the logger (°C) and RH was the relative humidity provided by the logger.

For the interpretation of results, first the average daily THI (values of 12.00 and 18.00 h) was estimated and then the severity of stress was assessed in the following way; <22.20: no heat stress, 22.20–23.29: moderate heat stress, 23.30–25.59: severe heat stress, ≥25.60: extreme severe heat stress [5].

#### 2.3.2. Reproductive Competence

Reproductive competence was assessed, as follows.

- Mating rate: number of ewes mated by a ram/number of ewes exposed to rams in each group × 100.
- Pregnancy rate: number of ewes diagnosed as pregnant on D73 / number of ewes mated in each group × 100.
- Lambing rate: number of ewes that lambed/number of ewes mated subsequently to oestrous synchronization in each group × 100.
- Total lambs born per ewe: number of liveborn and stillborn newborns/number of ewes that lambed in each group.

#### 2.3.3. Statistical Analysis

Data were analyzed by means of SPSS, version 13.0 (SPSS Inc., Chicago, IL, USA). Each value of *p* ≤ 0.05 was considered significant for all comparisons.

THI, breathing rate and rectal temperature were compared in group M or group C between the daily time points (12.00 and 18.00) by performing the paired samples *t*-test. Also, breathing rate and rectal temperature were compared between the groups M and C by performing the independent samples *t*-test.

Pregnancy rate on D73 and lambing rate between the groups M and C were compared by performing chi-square test. Number of total lambs born per ewe was compared by performing the independent samples *t*-test. The hematological variables and the cortisol concentrations were compared in group M or group C between the two time points (D33 and D40) by performing the paired samples *t*-test and between the groups M and C by performing the independent samples *t*-test.

The levels of TAC, GSH, and TBARS in time-series blood samples (D0, D33, D40) were compared in group M or group C and between the groups M and C by performing the general linear model for repeated measures. Each outcome was evaluated in an analysis of variance with time of blood sampling as a within-subjects factor and group as a between-subjects factor.

## 3. Results

### 3.1. Clinical Results—THI Estimation

**Figure 2:**
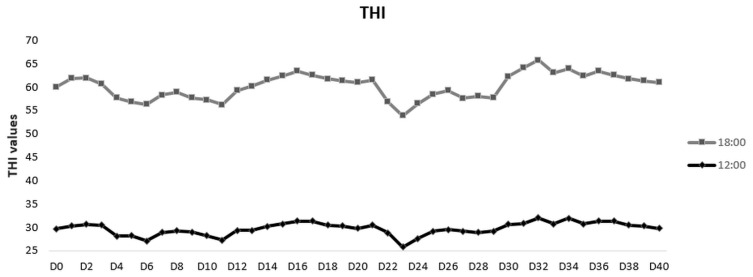
Temperature- Humidity Index (THI) throughout the study, estimated twice daily (12.00 and 18.00 h).

Mean THI throughout the experiment was lower (*p* < 0.001) in 12.00 (mean ± SEM: 27.5 ± 0.4) than in 18.00 (mean ± SEM: 32.7 ± 0.5) measurement. In all of the 80 occasions, extreme severe heat stress was estimated (Figure 2).

Mean rectal temperature and breathing rate (Table 1) were elevated at the 18.00 measurement (*p* ≤ 0.05 for all comparisons). Differences in average breathing rates and average temperatures throughout the experimental period were also significant between the groups M and C (*p* ≤ 0.05 for all comparisons).

### 3.2. Reproductive Performance

Following oestrous synchronization, all ewes were mated. The pregnancy diagnosis on D73 revealed higher number of pregnant ewes in group M (15/20–75.0%) compared to group C (9/20−45.0%) (*p* = 0.05), which finally lambed; pregnancy rate on D73 was equal to the lambing rate in each group. The number of total lambs born per ewe was higher in M group compared to C one (mean ± SEM: 1.73 ± 0.12 vs. 1.33 ± 0.17, respectively; *p* = 0.03).

### 3.3. Complete Blood Count

The mean values of measured hematological variables were within reference intervals [33]. For certain variables differences were found within time but not between groups. In both groups PCV values decreased significantly within time (Table 2, *p* ≤ 0.05). The number of total white blood cells (WBCs), neutrophils (NEU), and lymphocytes (LYMPH) decreased within time, but this decrease was found significant only in NEU number of group M (Table 2, *p* ≤ 0.05).

A decrease in NEU/LYMPH ratio was recorded in both groups within time, which did not differ between groups (Table 2, *p* > 0.05), but was significantly different within time in group M (Table 2, *p* ≤ 0.05).

### 3.4. Cortisol Measurement

The mean values of cortisol did not differ neither in group M or group C between the two time points (D33 and D40) nor between the groups M and C (Table 2, *p* > 0.05).

### 3.5. Redox Biomarkers

**Figure 3:**
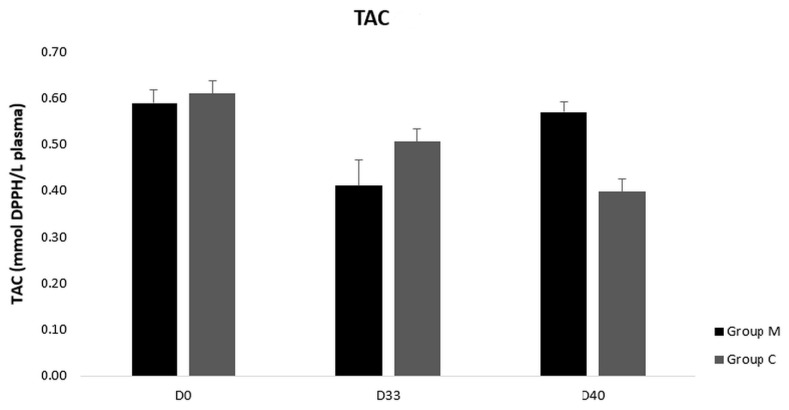
Total antioxidant capacity (TAC) in blood plasma of group M (melatonin) and group C (control) at three time points of the study. No difference was found between the two groups or between the three time points in group M or group C; *p* > 0.05.

**Figure 4:**
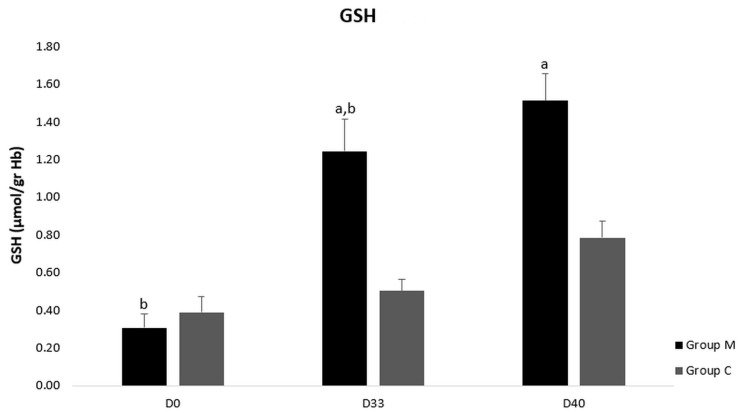
Glutathione (GSH) levels in blood plasma of group M (melatonin) and group C (control) at three time points of the study. a: Difference between the two groups, b: Difference between the three time points in group M or group C; *p* ≤ 0.05.

**Figure 5:**
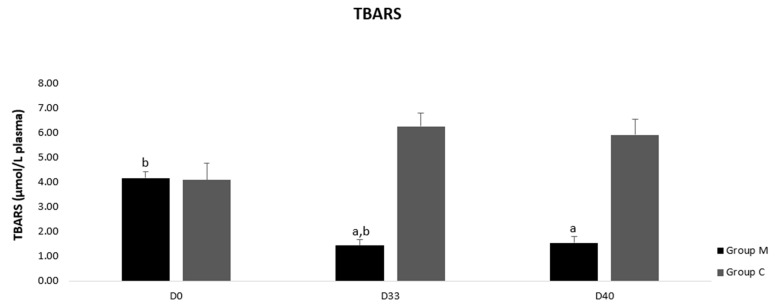
Thiobarbituric acid reactive substances (TBARS) levels in blood plasma of group M (melatonin) and group C (control) at three time points of the study. a: Difference between the two groups, b: Difference between the three time points in group M or group C; *p* ≤ 0.05.

TAC concentration did not differ neither between the groups M and C nor in group M or group C (*p* > 0.05) (Figure 3). However, GSH values were higher on D33 and D40 in group M compared to group C (*p* < 0.0005) (Figure 4). Accordingly, TBARS values were lower in group M compared to control one on D33 and D40 (*p* < 0.0005) (Figure 5). Moreover, GSH and TBARS values differed in group M between D1 and D33 or D40 (*p* = 0.002) but did not differ between D33 and D40 (*p* > 0.05).

## 4. Discussion

In this study the effects of melatonin administration, as an antioxidant regime, during the critical windows of preovulatory and preimplantation embryogenesis were evaluated in vivo, with respect to physiological adjustments and reproductive competence in heat stressed ewes. The results indicated increased fertility rate, improved mean number of lambs born per ewe, and enhanced redox status in ewes that received melatonin implants 34 days before the onset of oestruses, at a time when melatonin concentration was estimated to have reached its maximum level [22]. Τhe low cortisol values and the NEU/LYMPH ratio found in both groups may suggest that ewes of indigenous Karagouniko breed exhibit adaptation to environmental thermal stress, while melatonin administration seems to enhance it further.

THI was employed as an index of heat stress, as it represents a combined value of ambient temperature and humidity, for evaluating heat stress severity in farm animals [34]. Except for THI, rectal temperature and breathing rate are considered as heat stress indexes in sheep. The latter indexes indicated that all animals included in the study were imposed on extreme severe heat stress during the periconception and preimplantation period. All these physiological responses were evident in both groups reflecting the thermoregulatory adjustments to maintain homeostasis [35].

Heat stress effects on reproduction have been extensively reviewed [6]. Elevated temperatures may compromise the process of ovine oocyte maturation and other crucial cellular and molecular factors for embryogenesis. It is a fact that during the preimplantation developmental stages, the mammalian early embryos, especially at zygote stage, are rather susceptible to maternal elevated temperatures, leading to early embryonic loss [36,37].

The higher fertility rate found in melatonin treated ewes could be attributed to better redox status after administration of melatonin. High body temperatures caused under heat stress conditions enhance ROS generation that compromise fundamental cellular molecules, leading to cellular damage [38]. Early cleavage-stage embryos show high sensitivity to oxidative stress, because high ROS accumulation limits their developmental competence by causing cellular fragmentation and degeneration [39]. However, when heat stress was imposed at later stages of embryonic development, at the stage of 8–16 cells, at the morula or at the blastocyst stages, did not perturb the following formation of blastocysts [40]. A similar developmental competence of sheep embryos with regard to heat stress tolerance was also reported [36]. Thus, the low fertility rate in control group than in melatonin one could be attributed to the lower GSH and to the higher TBARS levels during the studied period.

The positive impact of melatonin on sheep embryos has also been proved and in our recent in vivo study, in which a positive correlation between exogenous melatonin, glutathione levels, pregnancy rate and number of lambs born was detected [25]. Melatonin into the follicular fluid acts as a scavenger of increased ROS, especially during the ovulatory process [41], and this melatonin action may explain the higher mean number of lambs born per ewe found in melatonin-treated group of the present study, as well. Numerous studies proved that adding melatonin in culture medium increased viability of sheep embryos [18,42]. Moreover, exogenous melatonin improved lamb production in sheep, at least in part, by improving the luteal function and embryonic survival [43]. Melatonin mediates for synchronizing the seasonal reproductive periods according to photoperiod and, besides, affects follicular growth and progesterone production by the corpora lutea in sheep [44]. The present study shows that the administration of melatonin as an antioxidant regime in heat stressed ewes could decrease thermal-oxidative stress during follicular growth, peri-conception period, and preimplantation stage embryos, thus improving the fertility and mean number of lambs born per ewe.

Melatonin provokes antioxidant effects at both physiological and pharmacological concentrations, by scavenging oxygen and nitrogen reactive species, too. This indolamine induces the synthesis of glutathione (GSH), which is an intracellular antioxidant and highly related with reduction of lipid peroxidation. This hormone has been proven to act as a regulatory factor in antioxidant gene expression, such as superoxide dismutase (SOD) and glutathione peroxidase (GPx). Regarding GPx, it seems that melatonin at low concentration cross-reacts with cell membrane and nuclear receptors, whereasat higher concentration it acts directly as a scavenger for radical species [17]. Probably, these actions are induced when melatonin interacts with the G protein-coupled transmembrane melatonin receptors MT1 and/or MT2 [45], which are present in several ovarian cell compartments [42,44].

In this study the M group showed better redox status compared to the control one. Specifically, GSH was higher and TBARS was lower indicating the antioxidant properties of melatonin. The lower lipid peroxide end product (i.e., MDA) and the higher GSH levels could probably be interpreted by a direct and/or an indirect antioxidant action of melatonin. Especially, glutathione, a scavenger of H_2_O_2_, is the major antioxidant component for scavenging ROS at oocyte level [7] and exerts a crucial role in embryonic developmental competence at preimplantation stages. When glutathione is added in IVM culture medium, the in vitro tolerance of bovine embryos to heat stress at 42 °C is increased [40]. As was indicated in mouse embryos [46], the harmful effect of heat stress is limited via glutathione-dependent mechanisms, while the enhanced redox balance may induce the development of thermotolerance. Moreover, as was reported in in vitro conditions in rat brains [47], pharmacological doses of melatonin caused a dose-dependent reduction in lipid peroxidation products (MDA and 4-hydroxyalkenals (CHDA)). Pregnant ewes under heat stress exhibit redox imbalance, as reactive oxygen metabolites accumulate and antioxidant mechanisms are hindered. Therefore, some antioxidants, such as melatonin, may reduce ROS levels in oocytes and early embryos by increasing glutathione levels.

Heat stress and elevated glucocorticoid levels were associated with embryo loss in Merino ewes. In the present study cortisol values did not show any difference neither between the two groups nor within each group during the period of study. The increase of cortisol levels in the systemic circulation has a positive effect on thermogenesis and a negative one on heat resistance. Cortisol concentration rises in animals exposed to elevated ambient temperatures, but progressively lessens when high temperatures persist for longer time periods [48]. The Karagouniko ewes participated in this study were exposed to high environmental temperatures almost for two months before the period that cortisol was evaluated. Presumably, the low levels of cortisol during extended period of heat stress reflect the animals’ adaptation and improved welfare to summer thermal conditions, as was previously suggested [49].

Hematological variables and leukocyte profile may provide information, on the degree of heat stress in animals. The present results of hematological analysis showed a significant decline of PCV within time in either melatonin-treated ewes or in control ones, but no difference was found comparing the two groups at two time points. These results are consistent with prior results [50,51,52,53] and contrary to others that found a significant increase in PCV [51]. In a recent study performed in heat stressed male lambs [50], the decline of PCV was counterbalanced by an increase in the size of erythrocytes and hemoglobin concentration in erythrocytes, which was interpreted as a mechanism of thermal adaptation. In the current study, the decline of PCV in both groups within time could be interpreted as a hemodilution effect, concerning the increase of water diffusion from interstitial fluid into the circulatory system, during the thermoregulatory process for evaporative cooling.

During the stress response, any change in the number and/or type of leukocytes is brought about to ensure their distribution according to the needs of the organism [54]. In the present study, no neutrophilia was observed, indicating no acute stress-response. On the contrary, the number of WBCs, as well as those of NEU and LYMPHs showed a decline, but remained within standard reference values within the one week that was studied in both groups, probably indicating an adaptation of Karagouniko sheep in heat stress conditions. Similarly, a lower number of WBCs was recorded in heat stressed animals [48,55]. However, in another study an increase in WBCs was found [48,56]. Lymphopenia may indicate an active stress response, too [54], as it is attributed to endogenous or exogenous corticosteroid actions [57]. In response to stress the decline in lymphocyte number is due to migration of lymphocytes from blood circulation into other tissues [54,58]. The significantly lower number of neutrophils found in melatonin group within time may indicate a better adaptation of melatonin ewes to heat stress. This is further supported by the significantly lower NEU/LUMPH ratio found in melatonin group within time, too. As was previously stated, melatonin may counteract the immunological depression induced by stress or glucororticoids [59]. Moreover, melatonin can down regulate the expression of glucocorticoids receptors [60], suggesting an inter-talk between melatonin and glucocorticoids in stressful situations. Notwithstanding, the NEU/LYMPH ratio represents a better indicator of stress response compared to the number of leukocytes or lymphocytes per se. An increase in NEU/LYMPH ratio is positively correlated with glucocorticoid secretion [54,61,62]. In this regard, a lower NEU/LYMPH was found in goats, suggesting a better adaptation to their environment [61].

## 5. Conclusions

This study ascertains, under in vivo conditions, that the prevention of the increase in oxidative stress originating from maternal elevated temperature may improve ovulation rate and the progress of preimplantation embryogenesis in sheep. Melatonin implants in ewes were found capable of inducing better fertility rate and higher mean number of lambs born due to improved redox balance. Although the low cortisol levels of the tested animals depict a progressive adaptation to environmental thermal stress, the implants seem to enhance this adaptation. In consonance with the need for sustainable solutions to the constantly budding climate change phenomena, our study suggests an alternative and novel tool for ameliorating the effect of heat stress, during the preovulatory period and early stages of embryogenesis, in ewes. Further evidence will shed light to a greater extent on this subject.

## Figures and Tables

**Table 1:** Rectal temperature and breathing rate (mean ± SEM) in group M (melatonin) and group C (control) during the week following the introduction of rams into the ewe flock (D34-D40).

Group	Time of Day	Days of Experimental Period						
		D34	D35	D36	D37	D38	D39	D40
Rectal temperature (°C)								
M	12.00	39.11 ± 0.05 ^a,A^	39.22 ± 0.05 ^a,A^	39.35 ± 0.04 ^a,A^	39.29 ± 0.04 ^a,A^	39.20 ± 0.05 ^a,A^	39.25 ± 0.05 ^a,A^	39.19 ± 0.05 ^a,A^
	18.00	39.72 ± 0.09 ^a,B^	39.95 ± 0.13 ^a^	39.91 ± 0.07 ^a^	40.06 ± 0.07 ^a,B^	40.09 ± 0.06 ^a^	40.01 ± 0.09 ^a^	40.17 ± 0.08 ^a^
C	12.00	39.27 ± 0.05 ^b,A^	39.24 ± 0.07 ^b,A^	39.20 ± 0.06 ^b,A^	39.27 ± 0.04 ^b,A^	39.35 ± 0.06 ^b,A^	39.37 ± 0.05 ^b,A^	39.36 ± 0.05 ^b,A^
	18.00	40.0 ± 0.03 ^b,B^	40.16 ± 0.02 ^b^	40.13 ± 0.02 ^b^	40.24 ± 0.02 ^b,B^	40.25 ± 0.03 ^b^	39.89 ± 0.03 ^b^	39.25 ± 0.02 ^b^
Breathing rate (breaths min^−1^, bpm)								
M	12.00	54.75 ± 2.8 ^c,C^	55.4 ± 2.3 ^c,C^	55.8 ± 2.2 ^c^	55.7 ± 1.7 ^c,C^	55.85 ± 1.5 ^c,C^	60.8 ± 1.6 ^c,C^	60.5 ± 2.1 ^c,C^
	18.00	89.05 ± 2.9 ^c,D^	82.75 ± 2.9 ^c,D^	92.1 ± 2.5 ^c,D^	87.35 ± 1.8 ^c,D^	89 ± 1.7 ^c,D^	95.4 ± 1.4 ^c,D^	95.5 ± 1.9 ^c,D^
C	12.00	65.6 ± 0.9 ^d,C^	65.9 ± 1.2 ^d,C^	65.7 ± 1.1 ^d^	66.4 ± 0.8 ^d,C^	68.7 ± 0.9 ^d,C^	67.1 ± 0.7 ^d,C^	68.1 ± 0.9 ^d,C^
	18.00	99.6 ± 1.2 ^d,D^	104.8 ± 1.7 ^d,D^	103.6 ± 2.1 ^d,D^	100.6 ± 0.9 ^d,D^	118.1 ± 4.2 ^d,D^	112.5 ± 1.9 ^d,D^	109.3 ± 2.2 ^d,D^

The same superscript ^a–d^ within the same column denotes significant difference (*p* ≤ 0.05). The same superscript ^A–D^ within the same column denotes significant difference (*p* ≤ 0.05).

**Table 2:** Hematological variables and cortisol concentration in blood plasma (mean ± SEM) in group M (melatonin) and group C (control) on D33 and D40.

Group Time		Hematological Variables								
		PCV	WBCs	NEU	LYMPH	MONO	EOS	PLT	NEU/LYMPH Ratio	Cortisol
		(%)	(cells/μL)	(cells/μL)	(cells/μL)	(cells/μL)	(cells/μL)	(cells ×10^3^/μL)		(μg/L)
**M**	**D33**	33.50 ± 0.80 ^a^	7956 ± 1140	3355 ± 467 ^c^	4814 ± 819	242 ± 53	345 ± 176	362 ± 27	0.73 ± 0.06 ^d^	1.23 ± 0.41
	**D40**	30.83 ± 0.96 ^a^	5467 ± 631	1584 ± 149 ^c^	3285 ± 341	152 ± 66	200 ± 94	356 ± 28	0.51 ± 0.06 ^d^	1.51 ± 0.36
**C**	**D33**	32.14 ± 0.84 ^b^	6529 ± 1665	1761 ± 197	2459 ± 952	102 ± 30	145 ± 77	382 ± 29	0.93 ± 0.28	0.71 ± 0.41
	**D40**	31.66 ± 0.98 ^b^	5643 ± 1287	1213 ± 179	2293 ± 687	123 ± 20	171 ± 25	370 ± 29	0.64 ± 0.23	0.59 ± 0.17

The same superscript ^a–d^ within the same group M or C denotes significant difference (*p* ≤ 0.05).

## Data Availability

The data could be provided on reasonable request from the corresponding author.
